# Predicting the potential of capacitive deionization for the separation of pH‐dependent organic molecules

**DOI:** 10.1002/elsc.202100037

**Published:** 2021-08-03

**Authors:** Robin Wagner, Sebastian Winger, Matthias Franzreb

**Affiliations:** ^1^ Institute of Functional Interfaces Karlsruhe Institute of Technology Karlsruhe Germany

**Keywords:** capacitive deionization, electrosorption, organic acid, pH dependence, product recovery

## Abstract

One of the main steps in the biotechnological production of chemical building blocks, such as, e.g. bio‐based succinic acid which is used for lubricants, cosmetics, food, and pharmaceuticals, is the isolation and purification of the target molecule. A new approach to isolate charged, bio‐based chemicals is by electrosorption onto carbon surfaces. In contrast to ion exchange, electrosorption does not require additional chemicals for elution and regeneration. However, while the electrosorption of inorganic salts is well understood and in commercial use, the knowledge about electrosorption of weak organic acids including the strong implications of the pH‐dependent dissociation and their affinity towards physical adsorption must be expanded. Here, we show a detailed discussion of the main pH‐dependent effects determining the achievable charge efficiencies and capacities. An explicit set of equations allows the fast prediction of the named key figures for constant voltage and constant current operation. The calculated and experimental results obtained for the electrosorption of maleic acid show that the potential‐free adsorption of differently protonated forms of the organic acid play a dominating role in the process. At pH 8 and a voltage threshold of 1.3 V, charge efficiencies of 25% and capacities around 40 mmol/kg could be reached for a constant current experiment. While this capacity is clearly below that of ion exchange resins, the required carbon materials are inexpensive and energy costs are only about 0.013 €/mol. Therefore, we anticipate that electrosorption has the potential to become an interesting alternative to conventional unit operations for the isolation of charged target molecules.

**Abbreviations**
CDI: capacitive deionization
CV: cyclovoltammetry
MA: maleic acid
PFL: potential free loading
SAC: electrosorption capacity

## 1 INTRODUCTION

Potential driven adsorption processes with porous carbon electrodes are well known for their application in water purification [1, 2, 3, 4, 5, 6, 7, 8, 9]. Due to the advantageous properties of the applied carbon materials, like low production costs, good conductivity and high inner surfaces corresponding with high adsorption capacities, even though not all of the surface is accessible to ions [1, 2], electrosorption is also of interest for other applications. One obvious application is waste water treatment [2, 8–19], as organic and inorganic chemicals can be found in industrial and municipal waste waters [8, 11]. A removal of these substances is necessary, since they can pollute natural water resources and have a harmful impact on ecosystems [8]. Another application which has been investigated is the purification or concentration of valuable charged molecules [9, 10, 18, 20–24]. Possible advantages in comparison to established thermal or chemical swing‐based separation technologies are the modularity and scalability of electrochemical processes [18, 25]. Another advantage is the simplicity of the regeneration step in comparison to ion‐exchange processes, where it often causes a loss of valuable products [18]. Especially in combination with a modification of the electrode surfaces, the efficiency and capacity of the electrosorption processes can be improved and an ion‐selectivity can be achieved [9, 25].

**FIGURE 1:**
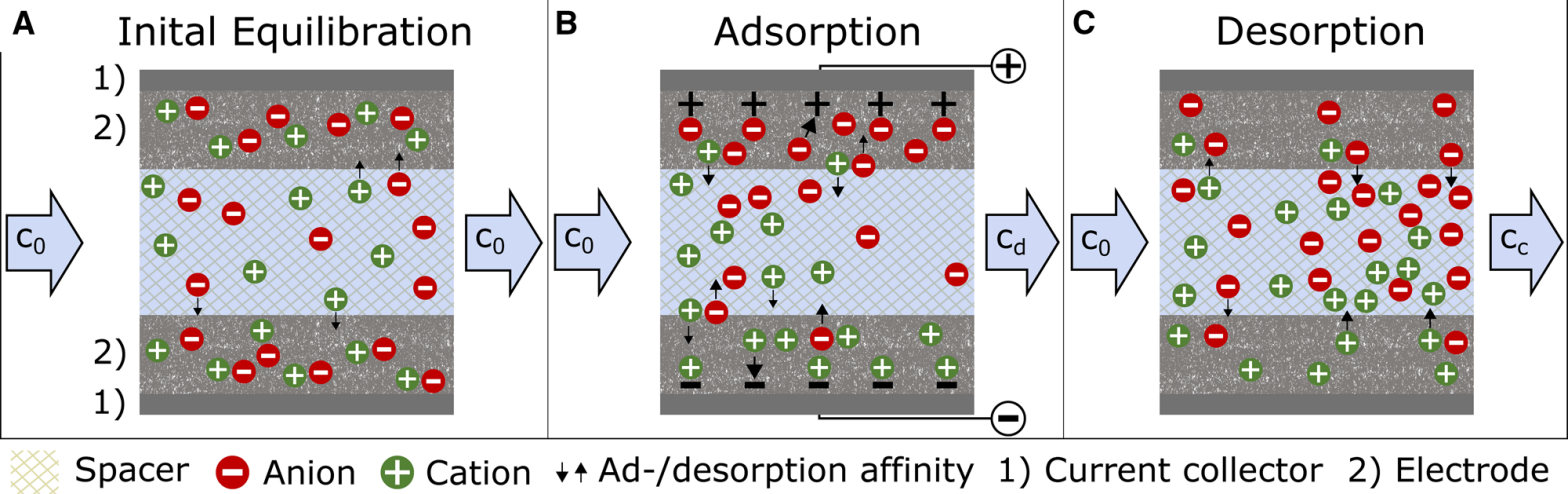
Schematic illustration of the ion distribution within a CDI system at different states of the process. (A) Initial equilibration without externally applied electrical potential; (B) Potential driven electrosorption; (C) Desorption caused by the removal of the external potential. (A) During the initial equilibration cations and anions accumulate equally in both electrodes due to physical adsorption and the condition of macroscopic electroneutrality. (B) By the application of an external electrical potential counterions are attracted to the oppositely charged electrode, while coions are pushed back into the solution. After some time counterion attraction starts to dominate and the solution in the middle channel gets depleted from ions. (C) If the potential is switched off, the process is reversed, resulting in a high concentration of desorbed ions in the effluent. At the same time, coions are adsorbed again and the system is re‐equilibrated

In a typical flow‐by electrosorption process, as shown schematically in Figure 1, an ionic solution flows through an electrochemical cell with at least two electrodes, which are placed in a short distance, only separated by a spacer with a thickness of approximately 100 to 300 μm [26]. In the initial phase of the process the electrode material is equilibrated with the feed solution without the application of an external voltage. During this equilibration phase species having a chemical affinity towards the carbon material of the electrode will accumulate in the region of the electrical double layer already, resulting in a potential‐free loading which will influence the following electrosorption step (Figure 1A). When a voltage is applied to the electrodes (up to a maximum of approx. 1.2 V because of water electrolysis [1]), the ions in the solution are attracted by the electrode with opposite charge and are stored in the electrical double layer (Figure 1B). However, at the same time ions in the electrical double layer having the same charge than the electrode will be repelled and will re‐enter in the bulk solution. If the uptake of ions prevails, their bulk concentration is reduced, resulting in an effluent showing a depletion in the charged target molecules. When the potential driven adsorption capacity is reached, a concentrated ion solution can be obtained by simply reducing the voltage to zero, implying the regeneration of the electrodes by the release of the ions (Figure 1C). For long regeneration times with continuous inflow of the feed solution, the system will re‐equilibrate into the situation described in Figure 1A. In case of a cyclic experiment complete regeneration rarely takes place. Rather, after several adsorption/desorption cycles, a cyclic steady‐state is reached in which just as much is adsorbed as is desorbed and one cycle resembles the other [27].

**PRACTICAL APPLICATION**

The experimental and calculated data presented can be used to assess the utility of static as well as dynamic electrosorption processes using the concept of capacitive deionization for separation of charged biological molecules. The introduced theory and its exemplary application offer a step by step approach, how the expected efficiencies and costs for the electrosorption of weak organic acids, such as maleic acid, can be determined. The approach requires only parameters which can be determined by simple batch experiments and fast electrochemical characterization techniques. Nevertheless, the influence of various parameters such as pH and concentration, as well as applied voltage and current onto key figures such as charge efficiency and electrosorption capacity is considered. By this, the theory and data will allow a fast decision in which cases electrosorption should be considered as a new alternative to conventional recovery steps of charged, small organic substances.

Potential free adsorption (often referred to as physical adsorption) is a major issue for the efficiency of electrosorption, and it can be expected that this is even more the case for organic molecules usually showing a higher affinity towards physical adsorption onto carbon materials than strong electrolytes like NaCl. The reason for the negative effect of this physically adsorbed ions on electrosorption processes becomes clear when one realizes that a charge entering the electrode can be balanced not only by the absorption of an oppositely charged ion, but also by the release of an equally charged coion, illustrated by the arrows in Figure 1B [1, 28–31]. Several attempts were made to decline the influence of coion repulsion by means of ion‐selective membranes placed in front of the electrodes [31, 32, 33]. Others modified the carbon of the electrodes itself to reduce the affinity towards physical adsorption [7, 29, 34]. Unfortunately, the use of membrane supported electrosorption does not work in case of charged organic molecules having four or more C‐atoms, because size exclusion hinders these ions from penetrating through the membrane. Therefore, a thorough understanding of potential‐free adsorption of charged organic molecules and its impact onto electrosorption is essential for an assessment of the potential of this unit operation for biotechnology.

During the last decade, the understanding and calculation of electrosorption, normally termed capacitive deionization in case of applications in the field of water treatment, made strong progress. This supported a targeted improvement of the materials used and the process parameters employed. There exist a variety of physiochemical theories based on the Gouy‐Chapman‐Stern model [5, 35, 36] or the modified Donnan model, which predict the accumulation of charged species in the electrical double layer or within micropores when an electrical potential is applied to a highly porous, conductive material. However, it quickly became evident, that the addition of a term considering chemical affinity between the ions and the carbon material is required for a satisfying description of experimental data. In most cases this is done by a constant term $\mu_{att}$ representing the attraction energy, or more precisely the difference of Gibbs free energy caused by the physical adsorption onto the carbon surface [37, 38, 39, 40, 41]. The approach has proven successful for low affinities and low concentrations; however, it also showed that a constant attraction energy overestimates the physical adsorption in case of higher concentrations in solution, because it does not describe any saturation effects [42]. In consequence, theories with variable $\mu_{att}$ have been developed. Biesheuvel et al. reported a model using an attraction energy which is inversely proportional to the ion concentration in the micropores [42].

Lately, the idea came up to simplify the prediction of capacitive processes when abandoning the modified Donnan model and replacing it by an extended dynamic Langmuir model [43]. For simplicity, the prediction of the temporal change in the system was omitted and instead the focus was placed on the relevant characteristic parameters resulting from the entire experiment. Nevertheless, also these models use terms to describe physical adsorption effects, which are not capable to capture pH‐dependent effects and the complex interplay of competitive multispecies adsorption.

On the experimental side, the main focus of studies investigating capacitive deionization was on the desalination of water, even though several studies also looked into potential‐controlled chromatography and electrosorption of small organic molecules relevant for biotechnological applications. Most of them also report the influence of the selected pH on the process and several explanations of the observed effects are given. Among these are the surface charge of the carbon [14], competition between hydroxide ions and the charged molecules [15], and the pH‐dependent dissociation of the molecules [44]. Other studies focused on the improvement of the separation by modifying the electrode surface [24], by using new carbon materials [23], or by optimizing the process set‐up and parameters [12, 22]. Besides the mentioned studies specifically investigating the effect of an electrical potential onto the sorption of organic molecules, there exist also more general studies about the potential‐free adsorption of charged organic molecules onto carbon surfaces. Several attempts were made to fit models based on the Langmuir isotherm to experimental data [45], sometimes with extensions to include multiple binding sites [46] or molecule interactions [47]. Others used the dilute solution theory to model the adsorption of organic molecules on carbon [48, 49], the Polanyi theory [50, 51], or calculations based on quantum mechanical density functional theory [52]. In most of these studies, the important effect of the pH is addressed and the models are formulated in a way which allows the consideration of pH variations [47, 53].

Our work aims to integrate these sophisticated multispecies theories of potential‐free adsorption into a CDI model, predicting the pH‐dependent efficiencies of electrosorption of organic acids based on only a few experiments and parameters specifying the carbon electrodes. Integrating the pH‐dependent dissociation equilibria of organic acids and the cooperative Moreau isotherm, we predict the potential‐free loading of CDI carbon electrodes over a broad pH and concentration range. The applicability of the resulting model and the selected genetic algorithm to determine the required model parameters was shown for other carbon materials and organic substances by Wagner et al. [47]. Combined with known correlations of the modified Donnan model, describing, e.g. the relationship between the resulting charge efficiency and the Donnan potential, a set of equations is derived predicting the electrosorption of weak organic acids with multiple deprotonation steps. The calculations are validated by experimental results obtained with a commercial lab scale CDI system and maleic acid as target molecule.

## 2 MATERIALS AND METHODS

### 2.1 Chemicals and materials

For all experiments, a modified Ecomite U (Pure Echem Ltd., South Korea) flow‐by CDI cell was used. The CDI cell was modified in a way that the ion exchange membranes in front of the electrodes had been removed, transferring the cell from a MCDI to a plain CDI unit. The cell has a normal two‐electrode arrangement with an inner volume of 10.8 mL. The volume and weight of the activated carbon layer of each electrode is 99 × 99 × 0.25 mm^3^ and 1.66 g. The electrodes are separated by a nylon cloth with a thickness 0.1 mm and a mesh size of 150 μm. The carbon layer material has a specific surface of 1692 m^2^/g, an average pore width of 1.9 nm, a porosity of 0.7 and a specific pore volume of 0.62 cm^3^/g. For a more detailed view of the topography of the electrode, a SEM image of a section of the electrode with a magnification of 2000x is included in the supporting information (SI) (SI 1.1 and Figure S3). For the specific capacitance ($C_{g}$) a value of 45 ± 0.16 F/g was determined by means of cyclovoltammetry (CV), measured with a 50 mM NaCl (≥99.5%, Merck, Darmstadt, Germany) solution and a scan rate of 0.1 mV/s for voltages between 0 and 0.2 V (details of the analysis of the CV data are described in SI 1.5).

The CDI cell contains one pair of electrodes, which are contacted with the feed in flow‐by mode. The solution enters the spacer filled gap between the electrodes at their circumference and leaves through a round recess in the middle of the electrode. In contrast to the original setup of the Ecomite U CDI cells, the ion exchange membranes were removed in order not to hinder the contact of the electrode material with larger organic molecules. As potentiostat a Gamry Reference 600 (Warminster, USA) was used. The flow rate was set with a Masterflex L/S peristaltic pump (Cole‐Parmer, Germany) and the measurement of pH and conductivity were conducted by a pH‐Meter 765 Calimatic (Knick, Germany) and a conductivity meter 703 (Knick, Germany). As probes connected to the measurement instruments, a SJ 114 (VWR, Germany) was used as pH‐electrode and a CDC‐314 (Radiometer Copenhagen, Denmark) as conductivity measuring cell. A picture of the entire setup and the CDI cell can be found in the SI (SI 1.1, Figures S1 and S2). The solutions were prepared with maleic acid, 99.0%, (Sigma Aldrich, USA), pK_a _= 1.92/6.23, molecular weight = 116.07 g/mol and for the adjustment of the pH a 2 M NaOH (Titripur, Merck, Darmstadt, Germany) was used.

### 2.2 Potential free adsorption onto the carbon material

For better comparability, also the measurement of the potential free loading was conducted in the CDI cell. For this, 50 mL of maleic acid solution (5 mM) adjusted to pH values between 4 and 9.5 by 2 M NaOH were pumped at 10 mL/min in a loop through the CDI cell until an equilibrium state was reached. Reaching the equilibrium state was assumed for constant pH values and conductivities measured by the sensors located behind the CDI cell. In order to determine the total concentration of dissolved maleic acid (independent of its dissociation state), calibrated measurements of the dissolved organic carbon (DOC) were used. For this, a sample of 4 mL of the solution was withdrawn and replaced with 4 mL of a 200 mM maleic acid (MA) solution and the same initial pH value. By this, a stepwise increase of the dissolved maleic acid was obtained and the corresponding potential free equilibrium loadings could be determined. The procedure resulted in equilibrium concentrations in a range of approximately 1‐60 mM. After the sixth equilibrium state was reached, the experiment was stopped. For the evaluation of the DOC results, we degassed the maleic acid samples with N_2_ for 5 min to remove dissolved CO_2_, making sure maleic acid is the only carbon‐based substance in the solution. The amount of adsorbed maleic acid (q) was calculated at first as the total initial amount of maleic acid ($c_{MA,0}\cdot V$) less the amount of maleic acid in solution after reaching the equilibrium state ($c_{MA,tot}^{*}\cdot V$) and divided by the electrode mass ($m_{E}$) (Equation 1)

(1) $$q_{1}=\frac{c_{MA,0}\cdot V-c_{MA,eq1}^{*}\cdot V}{m_{E}}$$

As described before, the concentration was increased by replacing 4 mL of the solution with a 200 mM maleic acid solution. The concentration resulting from this mixture was determined according to Equation (2).

(2) $$c_{MA,y}=\frac{c_{MA,y-1}\cdot \left(V-4\right)+c_{MA,concetrate}\cdot 4}{V}$$

For the following cycles (y = 2, 3, …6) the loading of the previous cycle had to be added (Equation 3).

(3) $$q_{y}=q_{y-1}+\frac{c_{MA,y}\cdot V-c_{MA,eq,y}^{*}\cdot V}{m_{E}}$$

### 2.3 Derivation of the adsorption isotherm

For the prediction of the potential free loading (PFL) of maleic acid onto the carbon material a model is required which considers the different dissociation states of maleic acid in dependence of pH and the varying affinities of these dissolved species for adsorption. In a recent publication, Wagner et al. [47] were able to show the suitability of an extended Moreau model [54, 55] to describe the adsorption of multi‐protonated organic acids onto different carbon materials, such as activated carbon and carbon nano tubes. The Moreau model belongs to the group of multispecies Langmuir isotherms with cooperative interaction between the adsorbed species. Therefore, we decided to use this model also for the description of potential‐free adsorption onto CDI electrodes and to determine the mutual interaction parameters of adsorbed species ($U_{XX}$), the adsorption affinities ($K_{X}$) and the maximal loading ($q_{max}$) of the carbon material by means of fitting these parameters to our experimental data applying a genetic algorithm. The subscript X represents the two charged dissociation stages of maleic acid (A, B), while later on A will refer to the monovalent species, B to the divalent species. Since non‐dissociated maleic acid is practically non‐existent in the investigated pH range and, at least in first approximation, also is not relevant for the electrosorption process, it is neglected in the following derivation.

For the case of a cooperative adsorption of two species A and B, the Moreau isotherm of species A can be written as:

(4) $$\frac{N_{A}}{N_{max}}=\frac{q_{A}}{q_{max}}=\frac{2K_{A}c_{A}+2{c_{A}}^{2}{K_{A}}^{2}e^{-\beta U_{AA}}+2c_{A}K_{A}c_{B}K_{B}e^{-\beta U_{AB}}}{1+2c_{A}K_{A}+{c_{A}}^{2}{K_{A}}^{2}e^{-\beta U_{AA}}+2c_{B}K_{B}+{c_{B}}^{2}{K_{B}}^{2}e^{-\beta U_{BB}}+2c_{A}K_{A}c_{B}K_{B}e^{-\beta U_{AB}}}$$

*N*_A_ and *N*_max_ represent the number of molecules A adsorbed to the carbon material and the maximal number of molecules, that can be adsorbed. Their ratio is therefore equal to the ratio of the loading of species A and the maximum loading. In the Moreau isotherm the loading depends on the concentrations of all species involved in the sorption process. Therefore, the pH value is considered inherently in the equation, since it is reflected by the degree of dissociation of the maleic acid. The total loading ($q_{ges}$) of maleic acid onto the carbon material can be expressed as the sum of the loading of all species

(5) $$\frac{q_{ges}}{q_{max}}=\frac{q_{A}+q_{B}}{q_{max}}=\frac{2K_{A}c_{A}+2{c_{A}}^{2}{K_{A}}^{2}e^{-\beta U_{AA}}+4c_{A}K_{A}c_{B}K_{B}e^{-\beta U_{AB}}+2K_{B}c_{B}+2{c_{B}}^{2}{K_{B}}^{2}e^{-\beta U_{BB}}}{1+2c_{A}K_{A}+{c_{A}}^{2}{K_{A}}^{2}e^{-\beta U_{AA}}+2c_{B}K_{B}+{c_{B}}^{2}{K_{B}}^{2}e^{-\beta U_{BB}}+2c_{A}K_{A}c_{B}K_{B}e^{-\beta U_{AB}}}$$

Finally, the potential free loading PFL, having a dimension of equivalents per gram as required in the expressions for predicting charge efficiencies of electrosorption, results from the loading of species A and B, weighted by their charges:

(6) $$PFL=z_{A}\cdot q_{A}+z_{B}\cdot q_{B}$$

According to the structure of a genetic algorithm [56, 57, 58], Equation 5 was used as the function, whose outcome ($q_{calc,i}$) is compared to the experimental results ($q_{exp,i})$. In addition, the coefficient of determination, Equation 7, was used as fit‐function [59, 60], with ${\bar{q}}_{exp}$ as mean value of the experimentally determined loadings.

(7) $$R^{2}=1-\frac{\sum_{i=1}^{N_{exp}}{\left(q_{calc,i}-q_{exp,i}\right)}^{2}}{\sum_{i=1}^{N_{exp}}{\left(q_{exp,i}-{\bar{q}}_{exp}\right)}^{2}}$$

The population size of the genetic algorithm was limited to 10 parameter sets in each iteration step. A steady‐state genetic algorithm was applied, where the best parameter set was kept unchanged for the following iteration step and the remaining nine parameter sets were generated as recombinations of the best five parameter sets. To preserve the possibility that all variables can take any value from the number range (0 to 10^5^ for adsorption affinities, 0 to 2.5 for $q_{max}$ and ‐30 to 30 for interaction parameters), the mutation rate of each parameter in the nine new sets was set to 20%. As break criterion, a limit of 200,000 was set for the number of iterations. For further calculations, mean values and standard deviations of 100 parameter optimization runs were used.

### 2.4 Electrosorption experiments

The electrosorption experiments were conducted with a flow rate of 2 mL/min in constant current mode with currents of 50 mA and 100 mA until a voltage of 1.3 V respectively 1.4 V was reached. After each electrosorption step, a constant voltage of 0 V was applied for 40 min to desorb the adsorbed ions. All experiments were conducted with an initial concentration of 10 mM maleic acid, but the solutions varied in pH.

During the electrosorption experiments the conductivity and the pH value were recorded on‐line for further evaluation. Since the measured conductivity represents the conductivity of all ions present in solution, it was necessary to combine this information with the pH data and the corresponding species distribution of maleic acid. In addition, the concentration dependent activity coefficients of the ions were considered by the correlation of Davies [61, 62]. By means of the pH value, the concentrations of hydroxy and hydrogen ions were determined and included into the calculations. Finally, the concentration of sodium ions can be calculated by means of the electroneutrality condition. The presence of sodium ions results from the addition of NaOH for pH‐adjustment. A detailed description of the derivation of species concentrations from conductivity and pH data is presented in SI 1.2.

Knowing the concentration of total maleic acid in the inlet of the CDI cell ($c_{0}$) and the outlet ($c_{out}$) during the electrosorption and desorption steps enables the calculation of the specific adsorption capacity SAC (for compatibility reasons with CDI theories for desalination we also use the term salt adsorption capacity, SAC) with Equation 8 under consideration of the flowrate ($\dot{V}$) and the mass ($m_{E}$) of both electrodes [63].

(8) $$SAC=\frac{\int \dot{V}\cdot \left(c_{0}-c_{out}\right)dt}{m_{E}}$$

To ensure a constant inflow concentration, a 2 L storage tank which was continuously stirred and gassed with nitrogen was utilized for the experiments. Due to the high volume of this tank, the effect of the CDI effluent which was returned back into the tank could be neglected. The charge efficiency Λ of the adsorption process was calculated as follows [63].

(9) $$\begin{array}{c} \Lambda =\frac{F\cdot SAC\cdot \left(\left|z_{-1}\right|\cdot \alpha +\left|z_{-2}\right|\cdot \left(1-\alpha \right)\right)\cdot m_{E}}{\int Idt}\cdot 100\% \end{array}$$

In Equation 8, it must be considered that especially in the pH range around the pKs of the organic acid, a mixed adsorption of mono‐ and divalent ions takes places. Therefore, the SAC value has to be multiplied by an average charge, resulting from the degree of dissociation α of the monovalent species. All shown electrosorption data are based on adsorption/desorption cycles, recorded in cyclic steady state, where each cycle is practically identical.

## 3 THEORY OF PH‐DEPENDENT ELECTROSORPTION OF WEAK ELECTROLYTES

### 3.1 Basic equations and boundary conditions

Predicting the double layer capacitance for fully overlapped electrical double layers, the modified Donnan model has proven to describe the electrosorption process in good agreement with experimental data [37, 38, 41]. According to this model, the micropore concentration $c_{i,mi}$ of an ion i is related to the concentration in the adjacent macropores $c_{i,ma}$ by:

(10) $$\begin{array}{c} c_{i,mi}=c_{i,ma}\cdot \exp\left(-z_{i}\cdot \varphi_{D}+\mu_{att}\right)\;\text{with}\;\varphi_{D}=\frac{\Delta \phi_{D}\cdot F}{R\cdot T} \end{array}$$

In this term $z_{i}$ is the charge of the ion and the dimensionless voltage $\varphi_{D}$ is determined by the Donnan potential $\Delta \phi_{D}$ normalized by the factor RT/F. In addition, the modified Donnan model includes a non‐electrostatic chemical attraction term $\mu_{att}$ which correlates with the potential free loading (PFL). In the following, we restrict ourselves to a fully dissociated 1:1 salt such as NaCl; however, the derivation will be extended later to the more general definition of PFL given in Section 2.3. In the case of a 1:1 salt, the electroneutrality condition enforces $c_{ma}^{+}=c_{ma}^{-}\;$for any location and time in the macropores. In addition, it is easy to recognize that the sum $\Delta c_{mi}\;$of the accumulation of counterions and the depletion of coions corresponds to the change in the ionic charge density in the micropores and therefore to the mass specific electric charge transfer within the CDI cell.

(11) $$\begin{array}{ccc} \Delta c_{mi} & = & \left(c_{Count,mi}-c_{0,mi}\right)+\left(c_{0,mi}-c_{Co,mi}\right)\\ & = & \Delta \sigma_{mi}=\frac{2\cdot \Sigma }{\;v_{m}} \end{array}$$

With $\Delta \sigma_{mi}$ being the change in the ionic charge density in mol/m^3^, Σ being the transferred charge per gram of electrode pair, and $v_{m}$ being the micropore volume per gram of electrode.

Inserting Equation 10 into Equation 11 and using the boundary condition that in case of a constant feed, having a salt concentration $c_{0}$, also in presence of an applied voltage the macropore concentration in equilibrium equals the feed concentration ($c_{0}=c_{0,ma}=c_{eq,ma})$, $\Delta c_{mi,eq}$ can be related to a corresponding equilibrium voltage $\varphi_{D,eq}$.

(12) $$\begin{array}{ccc} \Delta c_{mi,eq} & = & c_{0,ma}\cdot \left(e^{\varphi_{D,eq}}-e^{-\varphi_{D,eq}}\right)\cdot e^{\mu_{att}}\\ & = & 2\cdot c_{0,mi}\cdot \sinh\left(\varphi_{D.eq}\right) \end{array}$$

The derivation of this equation uses the fact that before the actual CDI experiment, saying during the equilibration phase of the setup without an applied potential ($\varphi_{D}=\;0)$, also in the micropores the concentration of the counter‐ and coions are equal and there is no net surface charge:

(13) $$c_{0,mi}=c_{0,ma}\cdot \exp\left(\mu_{att}\right)$$

At this point, we assume an ideal CDI process without any current losses by redox reactions at the electrode or non‐ideal isolators in the CDI‐cell.^1^ In this case, the total charge applied to the CDI cell will be used for counterion accumulation and coion depletion. This maximum charge amount applied can also be expressed in an equivalent, $\Delta c_{mi,max}$ achieved for a hypothetical maximal dimensionless Donnan potential $\varphi_{D,\max}$:

(14) $$\Delta c_{mi,max}=2\cdot c_{0,mi}\cdot \sinh\left(\varphi_{D,\max}\right)$$

### 3.2 Influence of the potential‐free loading onto charge efficiency for static systems

In principle, up to this point the introduced equations follow the derivation introduced by Biesheuvel, Porada, Levi, and Bazant for the modified Donnan model in case of a symmetric CDI cell fed by a 1:1 salt [42]. From the mentioned paper it can also be seen, that in this case, the charge efficiency Λ, defined as the ratio of adsorbed salt SAC and the applied charge Σ, is directly coupled to the dimensionless Donnan potential:

(15) $$\Lambda =\tanh\left|\frac{\varphi_{D}}{2}\right|$$

However, in [42] the dependence of $\varphi_{D}$ onto the material properties and the applied voltage is not explicitly given. By rearranging Equation 14 and substituting it into Equation 15, we derived an expression, coupling the charge efficiency to the initial ion concentration in the micropores before an external potential has been applied:

(16) $$\begin{array}{ccc} \Lambda & = & tanh\left|\frac{1}{2}\cdot arcsinh\left(\frac{\Delta c_{mi,max}}{2\cdot c_{0,mi}\;}\right)\right|\\ & = & tanh\left|\frac{1}{2}\cdot arcsinh\left(\frac{\Sigma }{v_{m}\cdot c_{0,mi}}\right)\right| \end{array}$$

While this correlation is included implicitly in the derivation of [42] already, Equation 16 allows the calculation of the expectable charge efficiency by a hand calculator and directly shows the disruptive influence of pronounced potential free adsorption effects. Extending the case of monovalent ions to the general case of a mixture of mono‐ and divalent ions in the micropores, $v_{m}\cdot c_{0,mi}$ must be replaced by the initial charge weighted micropore loading PFL. By this we get a direct connection between the total charge applied during the CDI process, the potential free loading of the micropores, and the resulting charge efficiency. Calculating the charge Σ by the simplified assumption of a constant mass specific capacitance $C_{g}$ of the electrodes and the potential difference (cell voltage) applied to the electrode pair $\Delta \varphi_{cell}$:

(17) $$\Sigma =\frac{1}{4}\cdot \Delta \varphi_{cell}\cdot \frac{C_{g}}{F}$$

the charge efficiency can finally be estimated by material data from literature or resulting from, e.g. CV measurements and simple, potential free, batch adsorption experiments.

(18) $$\Lambda =tanh\left|\frac{1}{2}\cdot arcsinh\left(\frac{C_{g}\cdot \Delta \varphi_{cell}}{4\cdot F\cdot PFL}\right)\right|$$

From Equation 18 it is easy to see that higher potential free loadings result in lower charge efficiencies and therefore also reduced salt adsorption capacities. Looking at Equation 18 it is important to realize, that the use of PFL for the description of the chemical affinity of the substances towards the electrode material is more general than the use of a constant $\mu_{att}$ in Equation 10. As already discussed in [42], constant values of $\mu_{att}$ in combination with higher feed concentrations, quickly result in unrealistically high potential free loadings. A possible approach to encounter this flaw is to introduce a $\mu_{att}$, which is inversely proportional to the concentration in the micropores, as it is done in [42]. However, in our case of applying CDI for the recovery of weak organic acids we encounter the pH‐dependent, cooperative adsorption of multiple species having different charges. Therefore, the derived equation for the charge efficiency contains PFL directly, with PFL being calculated by a cooperative Langmuir model (see Section 2.3). In case of higher feed concentrations, the PFL predicted by the cooperative Langmuir model approaches saturation, thus preventing unrealistically high potential free loadings.

### 3.3 Extension to dynamic and non‐ideal systems

As mentioned above, Equation 18 is based on the assumption of an ideal CDI process in a way that current losses by, e.g. redox reactions are neglected. If cell voltages above a critical value of approx. $\Delta \varphi_{crit}=1\;V$ are applied, this simplification may result in a significant overestimation of the charge efficiencies [64, 65] because parasitic currents occur. However, in first approximation, the effect of such non‐idealities can be taken into consideration by expressing the voltage‐dependent current, which is consumed for parasitic redox reactions, by a Tafel equation (Equation 19) [66, 67, 68].

(19) $$I_{redox}=a\cdot exp\left(\frac{\Delta \varphi_{cell}-\Delta \varphi_{crit}}{b}\right)$$

The parameters a,b can be determined by an independent experiment measuring the parasitic current of constant voltage experiments after equilibration of the CDI reaction (see SI 1.3, Figure S5). Knowing the total charge Σ supplied by the current source, as well as the estimated charge $Q_{redox}\;$spend by the redox reactions and calculated as the time integral of Equation 19 (see SI 1.3, Figure S4), the corrected charge efficiency is given by Equation 20:

(20) $$\Lambda_{corr}=\Lambda \cdot \left(1-\frac{Q_{redox}}{\Sigma }\right)$$

If the CDI cell is operated in constant current mode, as it was the case in the present investigation, a final effect must be considered. In constant current mode the system does not fully approach the equilibrium state corresponding to a given maximum voltage $\Delta \varphi_{pot}$ applied by the potentiostate, because the constant current I results in a constant potential drop in the current leads, carbon electrodes, and—at least in first approximation—in the electrolyte. When estimating the effective potential difference used for capacitive charging, these potential drops must be subtracted from the applied $\Delta \varphi_{pot}$.

(21) $$\Delta \varphi_{eff}=\Delta \varphi_{pot}-R_{setup}\cdot I-\frac{k_{cell}}{\kappa }\cdot I^{2}$$

with $R_{setup}$ being the combined ohmic resistance of the current leads and carbon electrodes, $k_{cell}$ being a cell constant in (A · m)^–1^, and κ being the conductance of the electrolyte in S/m. Knowing these resistances, $\Delta \varphi_{cell}$ in Equation 18 must be replaced by $\Delta \varphi_{eff}\;i$n case of constant current operation. For the used commercial CDI cell, we determined $R_{setup}=\;0.9\;\Omega$ and $k_{cell}=6/\left(A\cdot m\right)$ by resistance measurements of the current leads and the carbon electrode as well as of the full cell. The conductance depends on the concentration and type of the used feed solution. This is especially true for this work, investigating the applicability of CDI for weak organic acids, where the specific conductance also strongly depends on the degree of dissociation of the weak acid and therefore on the pH. Therefore, the pH‐dependent specific conductance is calculated using the known dissociation equilibria in combination with the Davies equation, describing the concentration dependence of the molar conductivities of the involved species [61, 62] (SI 1.2).

Once the charge efficiency is known, the amount of adsorbed salt (SAC) per gram of electrode pair can easily be calculated by:

(22) $$SAC=\Lambda_{corr}\cdot \Sigma$$

## 4 RESULTS AND DISCUSSION

### 4.1 Description of the physical adsorption of maleic acid by the Moreau isotherm

**FIGURE 2:**
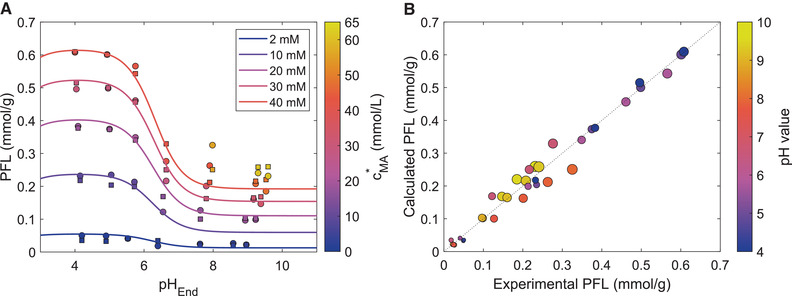
pH and concentration dependence of the potential‐free adsorption of maleic acid onto CDI carbon electrodes. (A) Potential‐free loading (PFL) of the electrode material after equilibration with maleic acid solutions of different pH and concentration. The squares mark the theoretical loadings predicted by the fitted Moreau isotherm model, the circles show the experimental data. The color of the markers is set according to the equilibrium concentration of the experiments. For a better guide of the eye, also the predicted trends of PFL versus pH for constant equilibrium concentrations of 2, 10, 20, 30, and 40 mM malic acid are added to the plot. From the data it can be seen, that in all cases the PFL shows a steep decrease around a pH value of 6.3, corresponding to second pKa2 of maleic acid. Therefore, the decrease in PFL correlates with the occurrence of the two‐times negatively charged maleic acid species, dominating in the pH range above pKa2. Comparing the PFL data along a vertical line (constant pH) it shows the common trend of increasing PFL with increasing equilibrium concentration. (B) Comparison of the calculated versus the experimentally determined PFL values. In case of a perfect fit all dots would arrange along the first bisector. It shows that the fitted Moreau isotherm allows a good fit throughout the investigated pH and concentration range. (Colormap: ametrine [73])

In the beginning of our study, we investigated the total loading ($q_{ges}$) of maleic acid onto carbon electrodes in a pH range between approx. 4 < pH < 10 and concentrations between approx. 1 and 60 mM after equilibration. In order to get comparable results, the adsorption experiments were conducted in the same CDI cell used for the potential driven experiments. The resulting loadings are shown in Figure 2 as circles filled with the color coresponding to the sum ($c_{Ma}^{*}$) of the equilibrium concentrations of all maleic acid species in solution.

For the evaluation of the results an extended Moreau model combined with a genetic algorithm was used, as it was described in the methods section. With the fitted model parameters the theoretical loadings were calculated for the same equilibrium conditions and plotted in Figure 2 as squares. The squares are also marked by the color code, allowing an easy identification of the corresponding pairs of experimental and calculated loadings. For illustation purposes, full isotherms for equilibrium concentrations of 40, 30, 20, 10 and 2 mM were added.

It should be emphasized, that the agreement between the experimental and the calculated loadings cannot be judged by the fact, if the markers closely follow the isotherms, since the resulting equilibrium concentration does not have to match any of the concentrations used for calculating the isotherms. The quality of the derived model is mainly visible by comparing the corresponding pairs of plotted points and squares, sharing the same equilibrium pH and concentration. The trend of the isotherms in Figure 2 shows an increasing loading for increasing equilibrium concentrations in solution, as it is typical for adsorption processes. The second trend which could be observed, is a decrease of the loading for pH values above approximately pH 5 leading to a constant loading for pH values above pH 8. A major reason for this trend is the pKa value of maleic acid of 6.23 and the resulting decreasing amount of monovalent maleic acid in solution with increasing pH. Since the adsorption affinity of the carbon material towards monovalent maleic acid is higher than its adsorption affinity towards divalent maleic acid species, the loading of the carbon material is strongly linked to the concentration of the monovalent maleic acid. A discussion of the influence of the carbon material on the pH shift occuring during the adsorption is given in SI 1.4.

The described dependencies are also reflected by the parameters determined for the Moreau model and listed in Table 1.

**TABLE 1:** Isotherm parameters of the Moreau model for maleic acid (MA) on EcomiteU carbon electrodes and the according standard deviation (SD)

Parameter	*K*_A_ (L/mol)	*K*_B_ (L/mol)	*q*_max_ (mmol/g)	*U*_AA_ (kJ/mol)	*U*_AB_ (kJ/mol)	*U*_BB_ (kJ/mol)	*R* ^2^
MA on Ecomite U	23.4 ± 0.26	5.3 ± 0.10	1.23 ± 0.01	‐0.36 ± 0.08	29.9 ± 0.02	3.04 ± 0.06	0.98 ± 0.00003

Both, the isotherm parameters and the standard deviation resulted as mean parameters of 100 calculations conducted with 200,000 iteration cycles.

Consistent with previous findings [49, 69], the affinity constant of the monovalent species (*K*_A_) is higher than the affinity constant of the divalent species (*K*_B_). The interaction parameters *U*_AB_ and *U*_BB_ are positive, corresponding to a repulsive force at the adsorption sites between mono‐ and divalent charged species or between two divalent species. In contrast, the value of *U*_AA_ is close to zero, implying there is almost no interaction between two adsorbed monovalent maleic acid molecules.

Based on the consistency between the experimental and the calculated loadings for a broad pH and concentration range, which is also reflected in the high *R^2^* of 0.98, it is reasonable to use the model with the parameters shown in Table 1 for the calculation of the PFL of the CDI electrodes, in order to investigate its influence on the adsorption capacity and the charge efficiency.

### 4.2 Electrosorption of maleic acid at varying pH

#### 4.2.1 Required input data and aim of the predictions

In the following, the charge efficiencies predicted by Equation 18 and the associated corrections for constant current operation mode and parasitic redox reactions will be compared to the experimental findings running a CDI cell with a feed solution of 10 mM maleic acid adjusted by NaOH to different pH values. The application of Equation 18 and the associated corrections do not need data of the CDI experiments, but some material data of the electrode and the feed solution, which can be gained from literature data or simple batch experiments. First, the parameters of the isotherm, describing the pH‐dependent preloading of the carbon material of the electrode are needed (see Section 4.1). Second, the electric capacitance of the electrode is needed. If this parameter is not delivered by the manufacturer or former experiments, a first approximation can be gained fast by chronoamperometry or cyclic voltammetry. Applying cyclic voltammetry, a value of 45 F/g was determined for the activated carbon electrodes used in the Ecomite‐U CDI cell. The respective cyclic voltammogram (Figure S7) and the way the mass specific capacity is derived is explained in detail in SI 1.5 of the supporting information. It should be mentioned, that the use of a constant value of the specific capacitance, independent of the applied voltage and the type and concentration of the electrolyte, is a rather strong simplification. However, the aim of our approach is to enable a fast evaluation of the applicability of CDI for the recovery of weak organic acids from, e.g. biotechnologically produced feedstocks, requiring a minimum amount of experimental data and only simple calculations, which can be done in Excel. At a later point, it would be possible without much effort to extent the derivation for, e.g. voltage‐dependent capacitances.

#### 4.2.2 Maximum electrosorption capacities in case of equilibrium, constant voltage conditions

**FIGURE 3:**
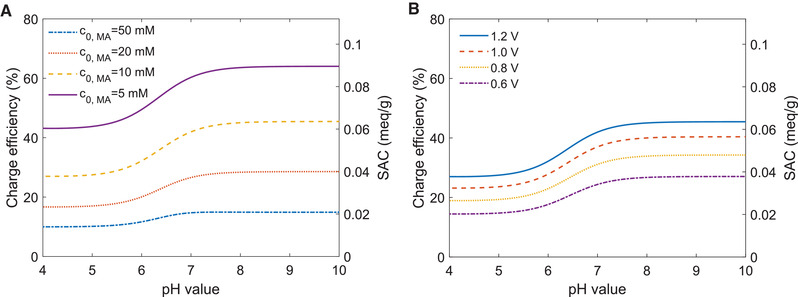
Predicted charge efficiencies and SACs for the electrosorption of maleic acid using a commercial CDI cell. The investigated operation conditions range from 4 < pH < 10, maleic acid concentrations between 5 and 50 mM, and applied cell voltages between 0.6 and 1.2 V. The calculations are conducted for idealized conditions, assuming a fully equilibrated system at a given cell voltage without any parasitic reactions consuming additional charge. Therefore, the values represent the maximum achievable charge efficiencies and SAC values. (A) Charge efficiencies and SAC values (applied cell voltage 1.2 V) show an increasing trend with increasing pH, while increasing concentrations result in a sharp decrease of both key figures. Also, in the case of salt adsorption capacities, the transition between low and high SAC values happens around the pH = pKa2. A comparison of Figures 2A and 3A shows that the PFL has a detrimental effect onto electrosorption and that high PFL values correspond to low SAC. This rule also explains the strong concentration dependence of the predicted SAC values. (B) pH‐dependent charge efficiencies and SAC values for different values of the applied potential (c_0,Ma_ = 10 mM). The plots show the expected increase of these key figures with increasing voltage. However, one has to keep in mind that in real system the effect of unwanted redox reactions at the electrode surface sharply increases above potentials of approx. 1 V

Knowing the parameters of the isotherm describing the potential free loading as well as the specific capacitance of the electrode material, at first, maximum expectable charge efficiencies in dependence of pH and maleic acid concentration can be calculated (see Figure 3). However, one has to be aware that these values are only valid in the case of constant voltage experiments approaching equilibrium and disregarding any non‐idealities, such as parasitic currents originating from redox‐reactions.

Plot A of Figure 3 illustrates the variation of the maximum charge efficiency in dependence of the solution pH and the total concentration of maleic acid. First, it shows that the pH has a pronounced effect, resulting in a significant increase of the charge efficiency from 42% up to 63% when changing the solution pH from 4 to 8 at a maleic acid concentration of 5 mM. Looking for the reasons of this increase, one has to keep in mind that at pH 4 the monovalent maleic acid species dominates, while at pH 8 almost all molecules of maleic acid are divalent. Therefore, a first thought could be that the increase is caused by the fact that divalent molecules show a stronger enrichment in the micropores when exposed to a potential difference (see Equation 10). However, a closer look quickly reveals, that at applied voltages of 0.6 V or higher, the thermodynamic driving force for the electrosorption of monovalent ions is more than enough to fully exhaust the available capacitance of the electrodes. To explain the increase, one has to look at Equation 18, which reveals that the achievable charge efficiencies are directly coupled to the PFL discussed in the previous subsection (see Figure 2) because the PFL strongly depends on the pH and shows a clear transition in the region of the second pKa (6.3) of maleic acid, these dependencies directly transfer to the achievable charge efficiencies and electrosorption capacities. As it is known from the extensive literature about adsorption of various organic substances onto activated carbons, the adsorption of strongly polar or charged substances is less favored compared to the adsorption of nonpolar substances. In addition, the adsorption of substances with multiple charges is less than the one of substances with only a single charge. In [47] Wagner et al. showed that Moreau isotherms are able to describe these dependencies of the PFL for different types of charged organic molecules over a wide range of pH and concentrations. In combination with the set of equations describing the dissociation equilibria of organic acids (see SI 1.2), the Moreau isotherm predicts a strong excess of dissolved divalent maleic acid species above pH 7 and consequently a much lower PFL within this pH range. In contrast, below around pH 5, the PFL easily exceeds the achievable ionic charge density of conventional CDI cells, which is about 0.2 mmol/g.

Besides the pH dependence, the PFL also shows a pronounced dependence onto the bulk concentration of maleic acid, although above 20 mM the onset of saturation effects gets visible. In comparison, concentrations above 5 mM are sufficient to fully explore the electrosorption capacities of CDI electrodes at voltages above 1 V. Therefore, as known from many other investigations about CDI of inorganic salts like NaCl, also in the case of somewhat larger organic molecules, charge efficiencies decrease with increasing concentration of the feed solution (see Figure 3A).

Looking at the plots of Figure 3A,B it shows, that the increased charge efficiencies directly transfer into higher salt adsorption capacities, when these are expressed in mol equivalents. However, when regarding the practical application of a CDI for the recovery of organic acids, the molar amount of maleic acid that can be bound onto the electrodes by electrosorption is more relevant. From the plot shown in the SI it becomes obvious that in the case of maleic acid adsorption using constant current operation until an equilibrium is reached, the increase in charge efficiency cannot compensate for the increased charge requirement of divalent maleic acid species (see SI 1.6, Figure S8). However, this situation may change for other organic species or other operation conditions.

#### 4.2.3 Electrosorption capacities in case of dynamic, constant current conditions

**FIGURE 4:**
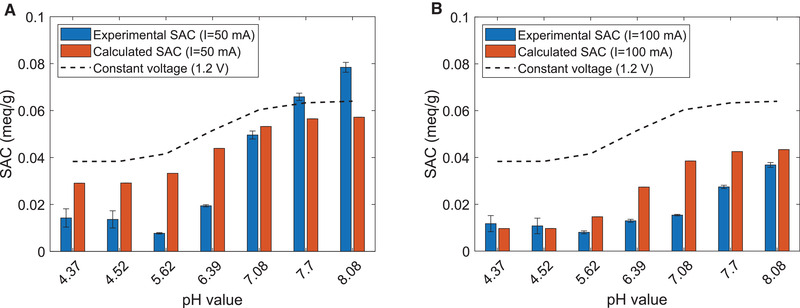
Experimental and predicted SAC values for the electrosorption of maleic acid applying dynamic CDI experiments conducted in constant current mode at 50 and 100 mA. c_0,Ma _= 10 mM, pH range 4‐8. The experimental values are determined from the third and following CDI cycles as a mean value of four cycles for each parameter set. A typical example of the cyclic concentration and pH profiles at this steady state operation can be seen in Figure 5. In the case of 50 and 100 mA, the electrosorption step was running until a cell voltage of 1.3 and 1.4 V, respectively. The increased cell voltage levels were chosen in order to compensate for the voltage drop in the current leads and electrode material occurring in constant current operation, with the aim to adjust a maximum potential difference of approx. 1.2 V between the electrode pair in both cases. A: SAC values for dynamic constant current experiments in case of an applied current of 50 mA. For comparison the trend of the maximum SAC values for idealized equilibrium conditions at a constant cell voltage of 1.2 V is also plotted as a dashed line. From the figure it can be seen, that also in the case of non‐idealized CDI operation including current induced potential drops and incipient, parasitic redox reactions, the pH dependence of the SAC values follows the discussed rules and shows a sharp increase around pH = pKa_2_. Interestingly the effect is even more pronounced in case of the experimental data, indicating additional contributions of, e.g. fixed functional groups at the carbon surface, which are not considered in our model. Nevertheless, the model gives a satisfying first approximation of the expectable magnitude and pH trend of SAC values in case of organic acids. (B) SAC values for dynamic constant current experiments in case of an applied current of 100 mA. After doubling the applied current, the measured and calculated SAC values show a strong decrease compared to the values at 50 mA, especially in case of higher pH values. The reason can be found in the increased potential drop in the electrolyte, reducing the potential difference which is effective for the electrosorption considerably

In order to extend the discussion to more dynamic constant current conditions and to validate the quality of these predictions, CDI experiments of a feed solution with 10 mM maleic acid were conducted in a pH range between 4 and 8, applying constant currents of 50 or 100 mA. As described in the theoretical section, the estimation of the achievable CDI adsorption capacities during constant current experiments is somewhat more complicated, because non‐equilibrium effects have to be considered. The most important effect is the one of the voltage drop occurring in the current leads but also in the electrolyte. For the conductive path running through the current lead to the carbon electrode, across the width of the electrode and back to the current source, we determined an ohmic resistance of 0.9 Ω. Therefore, based on the currents of 50 and 100 mA, a summarized voltage drop in these parts of the setup of 45 and 90 mV can be expected. While these values are constant for constant current operation, the voltage drop in the electrolyte is a function of its conductance and therefore of pH and concentration. Nevertheless, as shown in the supporting information, knowing the dissociation constants (SI 1.2) of maleic acid as well as the molar conductivities (SI 1.7) of the involved ionic species, a prediction of the resulting conductance is straightforward and achieves a high accuracy, when molar concentrations are corrected by the respective activity coefficients calculated by the Davies equation. Knowing its conductance, the voltage drop caused by the electrolyte can be estimated for different experimental parameters, after once determining the cell constant of the setup. Finally, the predicted electrosorption capacities in Figure 4 also consider the effect of parasitic redox reactions, which starts to get notable above cell voltages of about 1 V. However, as also shown in the supporting information, for the selected operation parameters, the effect of these parasitic reactions is rather small, being estimated to account for approx. 6% of the total current in case of 50 mA and approx. 3% in the case of 100 mA. Nevertheless, if higher cell voltages would be applied in an attempt to enhance the electrosorption capacity, these redox reactions will become a significant factor.

Taking into account the non‐equilibrium effects, Figure 4 shows that the suggested set of explicit equations is able to predict experimental results with sufficient accuracy. In assessing this statement, it should be considered, that predicting the adsorption capacity solely on the electric capacitance of the electrodes, without considering the effects of chemical affinity and voltage drop, the estimated electrosorption capacity at 1.2 V would be around 0.14 meq/g. In reality, the usable capacity for the electrosorption of organic acids is only a fraction of it. Normalized by the hypothetical maximum of the electrosorption capacity, the deviation between the predicted and estimated SAC is always less than 20%. Looking at Figure 4 in detail, the predicted pH dependence of the SAC follows the same trend as discussed for the static experiments in constant voltage mode. Also the experimental data follows this trend, however the differences between low and high pH values are even more pronounced. This manifests itself in the fact that at 50 mA and pH 8.1 the amount of captured molar equivalents of maleic acid is more than five times higher than in the case of 50 mA and pH 4.4. As a result, in the experiments the application of an increased pH turns out to be advantageous even if the molar amount of maleic acid bound by electrosorption is regarded. Another important fact revealed by the experimental results is that the application of a higher current of 100 mA strongly reduces the achievable SACs. Especially at higher pH values, the measured capacities at 100 mA are almost half the ones measured at 50 mA. Higher currents are associated with enhanced electrosorption rates, corresponding to lower residual concentrations in the bulk solution and the macropores of the electrode. The reduced concentrations are connected to lower conductivities and consequently a higher voltage drop in the electrolyte. In the end, the increased voltage drop has the consequence that the adjusted maximal cell voltage is approached quickly and the experiment is stopped before the full capacitance of the material could be used. These interdependencies are considered in the presented set of equations, allowing to correctly predict the strong influence of the applied current.

### 4.3 pH shift and lag phase during the electrosorption process

As can be expected, the potential driven ad‐ and desorption of different species of maleic acid, also results in pronounced changes of the measured pH value in the effluent of the cell. Because of the selectivities of the potential‐free but also the potential driven adsorption effects, the second dissociation equilibria of maleic acid in solution $HMA^{-}\;↔\;H^{+}+\;MA^{2-}\;$is disturbed. In consequence, monovalent maleic acid will deprotonate or divalent maleic acid will protonate until the equilibrium is readjusted, releasing or consuming $H^{+}$ ions. From Figure 2 it could be seen, that the chemical affinity of the electrode material strongly favors the physical adsorption of the monovalent species. In contrast, Equation 1 shows that the potential driven electrosorption results in a preferred accumulation of divalent counterions in the micropores. As described, the application of an electrical potential results on the one hand in a repulsion of preloaded maleic acids species (mainly monovalent) from the cathode into the solution. On the other hand, the anode accumulates maleic acid species in the micropores, however, with a preference for divalent species. Both processes result in an enrichment of the solution in monovalent species and a depletion in divalent species. In consequence, the shown dissociation equilibria reacts from left to right to counterbalance these processes, releasing $H^{+}$ ions by deprotonation of $HMA^{-}$ and thus decreasing the pH. This is exactly what can be observed in the experiment.

**FIGURE 5:**
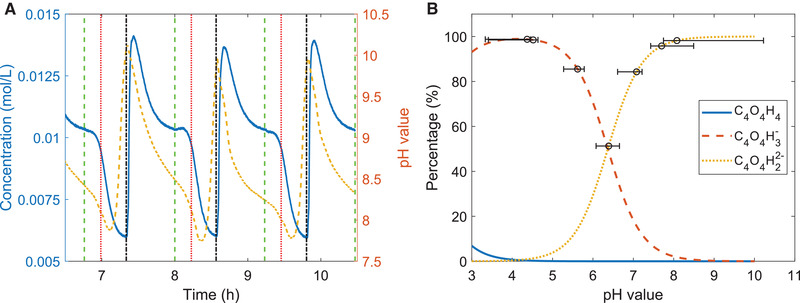
(A) Time course of concentration (blue line) and pH value (orange dashed line) during the adsorption (50 mA) and desorption (0 V) cycles. To indicate the beginning of the adsorption step, the time is marked by a green, dashed, vertical line and the beginning of the desorption step is marked by a black, dash‐dotted, vertical line. In addition, a red, dotted line is shown, which indicates the time when the amount of charge supplied during the adsorptions step equals the potential‐free loading. (B) Distribution of maleic acid species in solution for different pH values. The circles mark the pH value of the feed solution in different experiments. In addition, the whiskers indicate the extent of pH fluctuations observed in the effluent during ad‐ and desorption steps of the four cycles used for evaluation

Figure 5 shows the conductivity and pH profiles at pH 8.5 in the feed and a current of 50 mA during the electrosorption steps. While the current is applied, the pH constantly decreases below the pH of the feed solution. In contrast, when the current supply stops, the conductivity but also the pH show a steep rise, until pH values of about 10. At first sight, it may be unexpected that the pH shift to higher pH values is more pronounced than the one to pH values below the one of the feed (pH 8.5). However, the reason can be found in the buffer capacity of the maleic acid species. Figure 5B illustrates, that the pH changes are rather small in the pH‐region where mono‐ and divalent maleic acid species coexist in significant fractions. If only one species dominates to almost 100%, the pH shift gets much more pronounced and could stretch over more than two pH units.

In view of the shown importance of bulk solution pH onto the electrosorption of organic acids, it is important to discuss whether the conclusions made can be extended to the situation inside the micropores. As has been shown by, e.g. Dykstra et al. [70], according to the modified Donnan model, the pH in the micropores can vary from the one in the bulk by several units. However, such extreme differences can only be predicted in case of systems, in which the ion concentration in the bulk approaches very low values during the CDI process. This can easily be seen from the fact that the same Donnan potential must hold for all ionic species. Therefore, assuming that both, H^+^ and Na^+^, do not show non‐electrostatic chemical attraction, Equation 10 can be transformed to: $\frac{c_{H^{+},mi}}{c_{H^{+},ma}}=\frac{c_{Na^{+},mi}}{c_{Na^{+},ma}}$. This shows, that in case of an inlet concentration of 20 mM Na^+^ in the feed and a maximum ion concentration in the micropores of around 600 mM after applying a potential of, e.g. 1.2 V and equilibration with the feed, the maximum ratio of H^+^ between the micropores and the bulk is 30, corresponding to a pH‐difference of around 1.5. Comparing Equation 16 and Equation 18 it shows, that high potential free loadings further reduce the resulting Donnan potential and therefore the ratio of the concentration of a specific ion in the micro‐ and macro‐pores.

In case of our experiments, a maximum of $\varphi_{D}$ of 0.97 was calculated, corresponding to a rather small pH shift of 0.42 between the micro‐ and macropores. Although this is only a rough estimation, it shows that in case of the electrosorption of organic acids in a flow through CDI cell no extreme pH‐differences between micro‐ and macropores are to be expected. In this context should also be mentioned, that within the micropores the known dissociation constants of maleic acid species in free solution cannot be valid. The relationship between the micro‐ and macropore (bulk) concentrations is given by Equation 10. In addition, the macropore concentrations meet the constraints given by the dissociation equilibria. However, in this case the same dissociation equilibria cannot be fulfilled in the micropores.

Finally, another interesting detail, visible in Figure 5A, will be discussed. The plot shows the common drop in the effluent concentration when the current is applied. However, a closer look reveals that there is a significant delay of about 13 min between the moment the current is started (green, dashed marker) and the moment the concentration starts to drop in a steep descent. In good approximation, this delay corresponds with the hypothetical time t_E_ necessary for delivering a charge amount corresponding to the complete expulsion of the coions, which are re‐adsorbed to the carbon material during the potential‐free desorption steps.

(23) $$t_{E}=\frac{\mathrm{PFL}\cdot m_{E}\cdot F}{I}$$

The end of the time span *t*_E_ after the application of the current started is marked by a dotted, red line in all cycles. Again, the match between this simple estimation based on the potential free loading of the carbon material and the dynamic behavior of our CDI cell shows the importance of the understanding of the pH‐dependent adsorption phenomena for the prediction of the CDI performance.

## 5 CONCLUDING REMARKS

In this study, we show the influence of potential‐free adsorption on potential driven electrosorption processes for small organic molecules with multiple protonation states. We used an extended Moreau isotherm model including parameters for intermolecular interaction for the prediction of the potential free loading of the carbon material. The parameters were adapted to data from in situ experiments in a wide pH range between app. 4 and 10 and concentrations between 1 and 60 mM. Due to a high accordance of the model to the experiments, we were able to calculate the potential‐free loading at equilibrium and—in first approximation—at the end of the desorption steps of each potential driven experiment. Following, we showed the relation between this potential‐free loading, the SAC and the charge efficiency. For this, we derived a single equation which allows to predict the charge efficiency of constant voltage experiments reaching equilibrium conditions. In addition, we introduced extensions of the equation accounting for the inherent voltage drop during constant current operation and the reduction of charge efficiency resulting from parasitic redox reactions. Furthermore, the observed pH fluctuations during potential driven ad‐ and desorption cycles are explained at least qualitatively by the disturbance of the dissociation equilibria of dissolved maleic acid species caused by species selective potential‐free adsorption as well as electrosorption processes. These findings provide the opportunity to further optimize the electrosorption process of organic acids by selection of optimum combinations of electrode material, solution pH and operation conditions. What remains is the question, if the recovery of charged species of organic acids is limited by prohibitive energy and apparatus costs. Due to the lack of data for maleic acid, the following calculation is partly based on literature data on the fermentative production of fumaric acid [71], the isomer of malic acid in ‘trans’‐form. Assuming a fermenter of 10,000 L producing an organic acid at a productivity of 1 g/L/h the CDI step would have to handle 10 kg (86.2 mol) within 1 h. Based on a charge efficiency of 25% and energy costs of 0.1 €/kWh, this would result in operation costs of 1.1 €/h corresponding to 0.11 €/kg (0.013 €/mol) of product (see SI 1.8, SI). This simple calculation reflects the fact that during normal operation CDI cells only require electrical energy but no chemicals for elution or regeneration steps, comparable to filtration. A much more detailed cost estimation for CDI systems used in desalination, accounting, e.g. for capital costs and cyclic electrode replacement, has been published recently [72]. Reducing the NaCl concentration from 1.5 g/L in the feed stream down to 0.5 g/L in the effluent requires about 0.25 $/m^3^ (0.014 €/mol) in total for capital and operation costs. However, in case of desalination CDI charge efficiency is about twice the value we could reach in case of organic acids. Therefore, we can roughly expect that the cost will double in our case, reaching about 0.028 €/mol, corresponding to 0.25 €/kg for maleic acid. For comparison, maleic, fumaric, and succinic acid are rather cheap bulk chemicals, having a market price of about 1‐2 €/kg. This comparison shows that the costs for CDI are not prohibitive, but would account for a signification share of the product costs. Nevertheless, further improvements of CDI economics are likely because the process is commercialized only since a few years. A promising approach, which has been reported already by several groups but should be optimized with respect to organic target substances, is the introduction of functional groups at the carbon surface in order to reduce the chemical affinity between the carbon and the organic molecules. Therefore, we think in future CDI should be taken into consideration as a recovery step of charged, small organic substances, especially if precipitation steps rule out due to a high solubility of the respective substance, as it is the case for maleic acid.

## NOMENCLATURE

**Table:** 

Symbol	Unit	Description
a	A	Exchange current
b	V	Tafel equation parameter
c	$mol\cdot L^{-1}$	Concentration
$C_{g}$	$F\cdot g^{-1}$	Specific capacitance
F	$96485\;A\cdot s\cdot mol^{-1}$	Faraday constant
I	A	Current
$I_{redox}$	A	Redox‐Current
K	$L\cdot mol^{-1}$	Binding affinity
$k_{cell}$	$A^{-1}\cdot m^{-1}$	Cell constant
$m_{E}$	g	Electrode mass
N	mol	Number of molecules
PFL	$eq\cdot g^{-1}$	Potential free loading
Q	C	Charge
q	$mmol\cdot g^{-1}$	Loading
R	$8.314\;V\cdot A\cdot s\cdot mol^{-1}\cdot K^{-1}$	Gas constant
$R^{2}$		Coefficient of determination
$R_{setup}$	Ω	Resistance of the setup
SAC	$mol\cdot g^{-1}$	Salt adsorption capacity
$t_{E}$	s	Expulsion time
U	$kJ\cdot mol^{-1}$	Interaction parameter
V	L	Volume
$V_{cell}$	V	Cell voltage

Greek symbol	Unit	Description
β	J	Boltzmann factor
Λ	%	Charge efficiency
$\mu_{att}$		Chemical attraction term
$\nu_{m}$	$m^{3}\cdot g^{-1}$	Micropore volume
σ	$mol\cdot g^{-1}$	Ionic charge density
Σ	$eq\cdot g^{-1}$	Transfered charge
$\varphi_{D}$		Dimensonless voltage
$\Phi_{D}$	V	Donnan Potential
κ	$S\cdot m^{-1}$	Electrolyte conductance

## CONFLICT OF INTEREST

The authors have declared no conflict of interest.

## Supporting information

Supporting Information

## Data Availability

The data that support the findings of this study are available from the corresponding author upon reasonable request.

## Footnotes

1 The effect of additional current loses will be considered at a later point.
