# Supplementary material for: Predicting the potential of capacitive deionization for the separation of pH‐dependent organic molecules
Source: Eng Life Sci. 2021 Aug 3;21(10):589–606. doi: 10.1002/elsc.202100037 (PMC8518579; doi:10.1002/elsc.202100037)
Supplement: Supplementary file 1 — Supporting Information [file ELSC-21-589-s001.pdf]

SI 1. Supporting Information

SI 1.1 Materials and Methods

The experimental setup including the analyzing equipment (marked as 1,2,6,7), the 2 L storage tank and the magnetic stirrer (3 and 8), the peristaltic pump (4), the potentiostat (5) and the CDI-unit (9) is shown in Figure S 1.

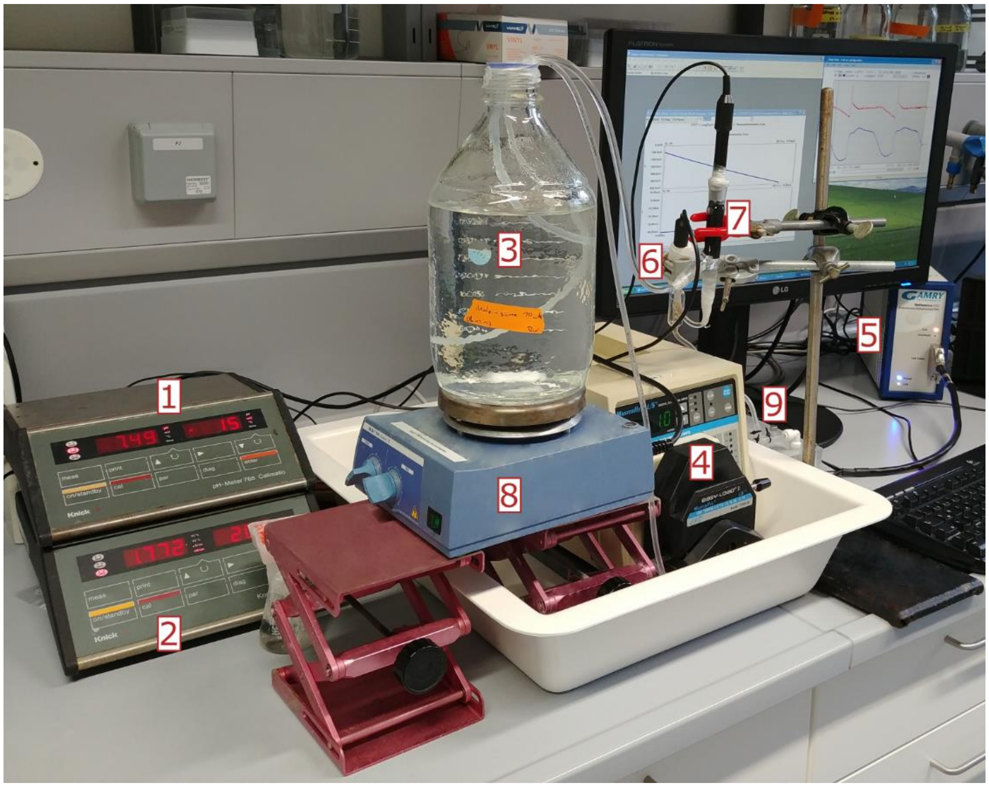

Figure S 1: Setup for electrochemical adsorption experiments. 1: pH-Meter, 2: Conductometer, 3: Solution Reservoir, 4: Peristaltic pump, 5: Potentiostat, 6: Conductivity cell, 7: pH-probe, 8: Magnetic stirrer, 9: CDI-unit

Since the CDI-unit is the central element in the experimental setup, a close-up photography of it is presented in Figure S 2. Since the conductive graphite sheet carrying the activated carbon layer is sensitive to mechanical impact, a bracket for the CDI-unit was 3D printed. The bracket allows the electrode to be contacted by a copper plate to which the potentiostat can be connected.

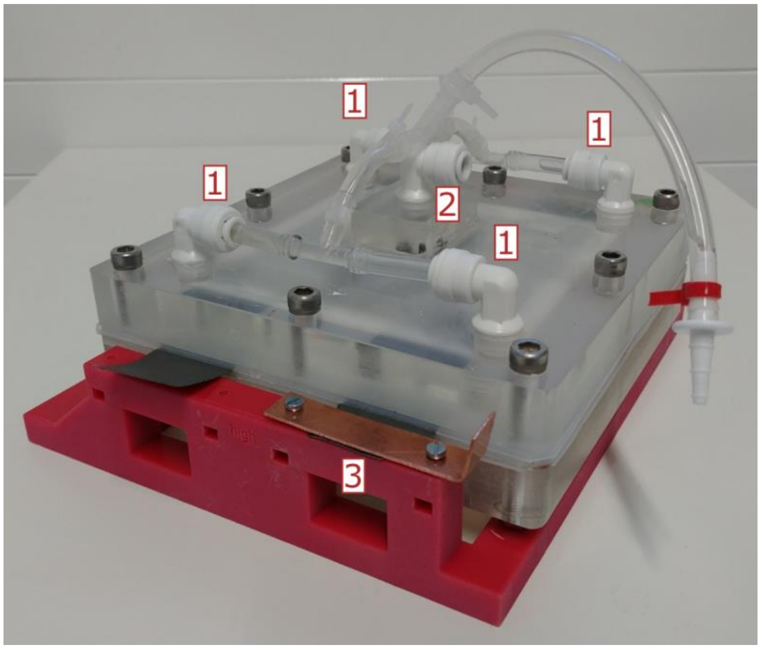

Figure S 2: CDI-unit Ecomite U in a 3D-printed bracket to enable the contacting of the conductive support layer of the electrodes via a copper sheet. 1: Inlet of the CDI-unit, 2: Outlet of the CDI-unit, 3: Contact of an electrode contacted with a copper sheet.

An insight into the morphology of the activated carbon layer of the electrode is given through Figure S 3, where an image of the electrode surface is presented, taken with a scanning electron microscope with 2000x magnification.

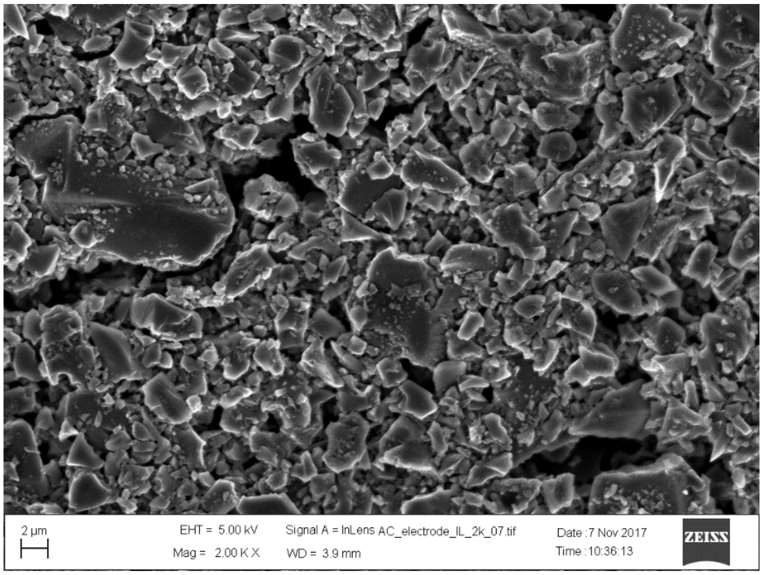

Figure S 3: Image of the surface of an electrode used in the CDI-unit Ecomite U (Pure Echem Ltd., South Korea) taken with a scanning electron microscope with 2000x magnification.

The surface of the electrodes is rough, has some fractures and seems not to be covered with the binder, which can be useful in terms of mass transport due to the accessibility of macro- and micropores.

SI 1.2 Determination of the concentration of maleic acid from pH and conductivity

The following comprehensive section describes the general approach, how the pH-dependent fractions of the different species of a weak organic acid with two deprotonation steps can be calculated. However, as explained in the main text, for the investigated pH range an iterative process was used to determine the concentration of maleic acid from pH and conductivity data measured online. At the beginning, the ionic strength is estimated by the feed or initial batch concentrations.

$$I_S = \frac{1}{2}\sum c_i z_i^2 \tag{1}$$

In the following iterations, the ionic strength of the previous step is used. According to the ion product of water, the activities of OH^- and H_3O^+ were determined by the measured pH value (equations (2)) [1]:

$$a(H_3O^+) = 10^{-pH} and\ a(OH^-) = 10^{-(14-pH)} \tag{2}$$

By the help of the ionic strength, the activity coefficients of monovalent and divalent ionic species can be calculated according to the Davies equation [2], [3]:

$$lg(\gamma_i) = -0.5 \cdot z_i^2 \left(\frac{\sqrt{I_S}}{1+\sqrt{I_S}} - 0.3 \cdot I_S \right) \tag{3}$$

From this equation we find: $\gamma_+ = \gamma_- = \gamma_1$ and $\gamma_{2-} = \gamma_2$, while the activity coefficient of the neutral species can be set to 1 in the examined concentration range. The activity coefficients are required, because while the equilibrium constants of the two dissociation reactions, Ks_1 and Ks_2, are defined using the activities of the species, the degree of dissociation α_1 and α_2 of the neutral and monovalent species is defined using molar concentrations. By the help of the activity coefficients the following relationships between the thermodynamic equilibrium constants defined by activities and hypothetical equilibrium constants defined by concentrations can be derived:

$K_{s1}^c = \frac{K_{s1}}{\gamma_1^2}\ \ and\ K_{s2}^c = \frac{K_{s2}}{\gamma_2}$

Using these relationships and the following definition of the degrees of dissociation of a two-protonic weak acid we find:

$$\alpha_1 = \frac{K_{s1}^c}{K_{s1}^c + [H_3O^+]}\ and\ \alpha_2 = \frac{K_{s2}^c}{K_{s2}^c + [H_3O^+]} \tag{4}$$

with $[H_3O^+] = \frac{a(H_3O^+)}{\gamma_1}$

These degrees of dissociation are independent of the total concentration of maleic acid present. Solving the mass balances for the two reactions gives the relationship between the concentrations of the three dissociation stages and the degrees of dissociation in the following by equations (5) (6) (7):

$$[C_4O_4H_2^{2-}] = \frac{\alpha_1 \cdot \alpha_2}{(1 - \alpha_2 + \alpha_1 \cdot \alpha_2)} \cdot c_0 \tag{5}$$

$$[C_4O_4H_3^{-}] = \frac{\alpha_1 \cdot (1 - \alpha_2)}{(1 - \alpha_2 + \alpha_1 \cdot \alpha_2)} \cdot c_0 \tag{6}$$

$$[C_4O_4H_4] = \frac{(1 - \alpha_1) \cdot (1 - \alpha_2)}{(1 - \alpha_2 + \alpha_1 \cdot \alpha_2)} \cdot c_0 \tag{7}$$

The electroneutrality in the solution is used to calculate the concentration of sodium ions in the solution, which can be found due to the usage of sodium hydroxide as base.

$$[Na^+] = [OH^-] - [H_3O^+] + [C_4O_4H_3^-] + 2 \cdot [C_4O_4H_2^{2-}] \tag{8}$$

Finally, with all the concentrations of the species determined, the specific conductivity of the solution can be calculated as the sum of the specific conductivities of all i species (see also next section):

$$\kappa = \sum_i \Lambda_i \cdot \gamma_i \cdot c_i \tag{9}$$

The specific conductivity is defined as the product of the concentration of each species, corrected by the activity, multiplied by the equivalent conductivity $\Lambda_i \left(\frac{cm^2}{\Omega \cdot mol}\right)$ [4].

In the end, the calculated conductivity is compared with the measured conductivity. If the difference between the two values exceeds a given tolerance, the assumed total concentration of maleic acid species in solution is adjusted accordingly and a new step of the iterative algorithm is conducted.

SI 1.3 Influence of parasitic redox reactions onto charge efficiency

The total current (I_{total}) measured by the potentiostate is the sum of the effective current used for capacitive adsorption (I_{cap}) and the current which is consumed due to redox reactions (I_{redox}), as shown in Figure S 4.

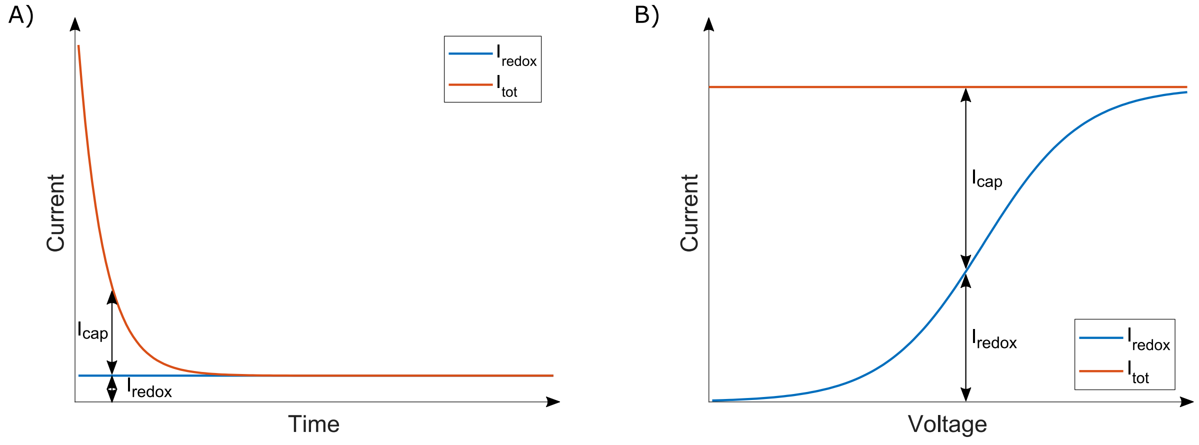

Figure S 4: Shown are the currents resulting for A) constant voltage experiments and B) constant current experiments. The measured current is a combination of the current resulting from capacitive effects and the current, lost due to redox reactions.

The redox current I_{redox} was calculated by the Tafel equation [4]–[6].

$$I_{redox} = a \cdot exp\left(\frac{\alpha_A \cdot F \cdot (V_{cell} - V_{crit})}{R \cdot T}\right) \tag{10}$$

With a, an exchange current, dependent on temperature and concentration, α_A as charge transfer coefficient, F as Faraday constant in As/mol, R as ideal gas constant in J/(mol*K), T the temperature in K. Replacing $\frac{R \cdot T}{\alpha_A \cdot F}$ by the constant b:

$$I_{redox} = a \cdot exp\left(\frac{V_{cell} - V_{crit}}{b}\right) \tag{11}$$

The current I_{cap} is coupledto the amount of ions entering and leaving the double layer. Therefore, the correlation between the voltage change over time and the current I_{cap} is related to the capacity C_{cell} of the CDI cell and is given by:

$$\frac{dV(t)}{dt} = \frac{1}{C_{cell}} \cdot I_{cap} \tag{12}$$

Replacing I_{cap} by difference of I_{tot} and I_{redox} we get:

$$\frac{dV(t)}{dt} = \frac{1}{C_{cell}} \cdot (I_{tot} - I_{redox}) = \frac{I_{tot}}{C_{cell}} - \frac{I_{redox}}{C_{cell}} \tag{13}$$

With $d = \frac{I_{tot}}{C_{cell}}$ and $k = \frac{a}{C_{cell}}$ as well as replacing I_{redox} by equation (11), equation (13) can be written as:

$$\frac{dV(t)}{dt} = d - k \cdot exp\left(\frac{V_{cell} - V_{crit}}{b}\right) \tag{14}$$

Solving this differential equation and applying the boundary condition $V(0) = 0$ we get:

$$V(t) = V_{crit} - b \cdot ln\left(\frac{\exp\left(-\frac{d \cdot t}{b}\right) \cdot \left(d \cdot \exp\left(\frac{V_{crit}}{b}\right) - k\right) + k}{d}\right) \tag{15}$$

Replacing V of the Tafel equation by $V(t)$, we get a time dependence for $I_{redox}(t)$:

$$I_{redox}(t) = a \cdot \frac{d}{k + \exp\left(-\frac{d \cdot t}{b}\right)\left(-k + d \cdot \exp\left(\frac{V_{crit}}{b}\right)\right)} \tag{16}$$

By integrating the redox current (eq.(16)) for the adsorption period from t=0 to t_{end}, the amount of charge lost due to faradaic reactions can be determined.

$$Q_{redox}(t) = C_{cell} \cdot b\left(ln\left(k \cdot \left(exp\left(\frac{d \cdot t}{b}\right) - 1\right) + d \cdot exp\left(\frac{V_{crit}}{b}\right)\right) - ln\left(d \cdot exp\left(\frac{V_{crit}}{b}\right)\right)\right) \tag{17}$$

To determine t_{end}, the maximum voltage can be set into equation (15) after solving it for the variable t, as shown in equation (18)

$$t = \frac{b}{d} \cdot ln\left(\frac{d \cdot exp\left(\frac{V_{crit}}{b}\right) - k}{d \cdot exp\left(\frac{V_{crit}}{b} - \frac{V}{b}\right) - k}\right) \tag{18}$$

Therefore, the charge efficiency can be calculated as:

$$\Lambda = \Lambda_{id} \cdot \left(1 - \frac{Q_{redox}(t_{end})}{Q_{ges}}\right) = \Lambda_{id} \cdot \left(1 - \frac{Q_r(t_{end})}{I_{ges} \cdot t_{end}}\right) \tag{19}$$

In order to determine parameters a and b of the Tafel equation, an experiment was conducted with stepwise increasing voltages from 0.1 to 1.2 V. Two liters of a maleic acid solution were prepared with a concentration of 10 mM and pH 8.5 after the addition of NaOH. After degassing it with nitrogen, it was pumped through the CDI-unit at a flow rate of 2 mL/min for 3600s. At this time the remaining current was registered and plotted versus the applied voltage. The resulting plot I_{redox} versus V was used to fit the Tafel equation (eq. (11)).

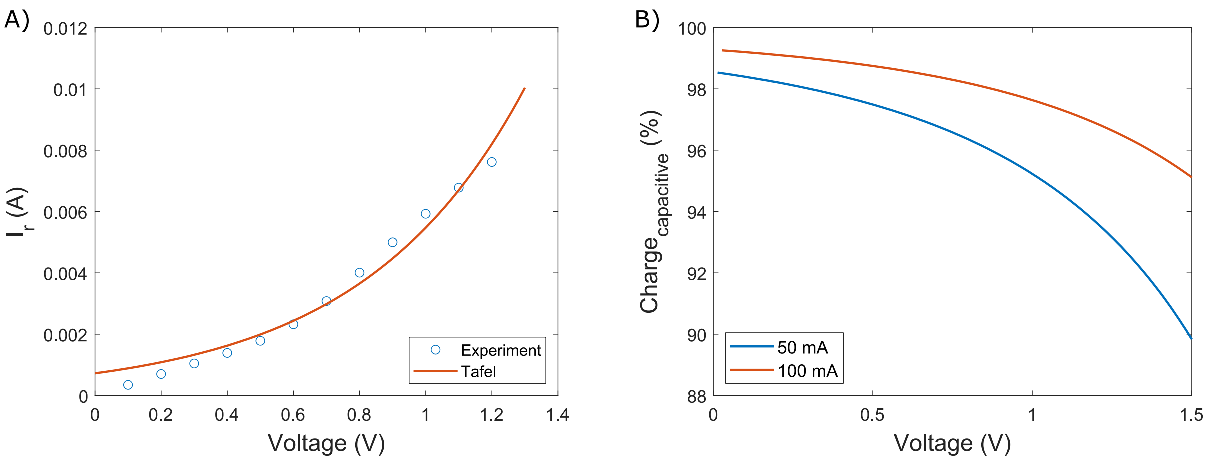

Figure S 5: A) Experimentally determined residual redox currents for constant voltage CDI experiments reaching equilibrium state of the capacitive processes after 3600s. Feed solution: 10 mM maleic acid, pH 8.5. The red line shows the fit of the experiments conducted at different voltages to the Tafel equation. B) Fraction of the total charge supplied which is used for capacitive effects. The fraction is calculated for constant current CDI experiments in dependence of the maximum voltage allowed before the experiment is stopped.

As shown in Figure S 5 A, the fit with the Tafel equation shows a good agreement to the experimentally determined redox currents. The determined parameters are a=0.0055 A and b= 0.495 V, for V_{crit}=1 V. In Figure 5B the amount of charge used for electrosorption is shown as fraction of the absolute charge for constant current experiments at 50 and 100 mA. The voltage on the x-axis shows the maximum voltage at which the constant current supply is stopped. The charge used for capacitive electrosorption was determined by subtracting C_{redox} (eq. (17)) from the total charge flown until the time/voltage of interest. The resulting fraction was used as a correction factor for the prediction of the charge efficiency including losses due to redox reactions.

SI 1.4 Influence of carbon on the pH shift

In the potential-free adsorption experiments of maleic acid onto the electrode material of the CDI system we observed pronounced pH shifts during the step-wise addition of the maleic acid solution adjusted to a certain pH. Except for the experiment conducted at the highest pH, the pH of the solution circulated in the loop quickly raised after the injection of maleic acid, although the injected solution was adjusted to the aspired pH in advance. The reason for the pH shift during the adsorption is mainly the higher affinity of the carbon material towards non-charged or monovalent maleic acid species. Together with the varying buffer strength of dissolved maleic acid species at different pH, this results in the trend of the pH shift

shown in Figure S 6. Details on the prediction of the pH shift resulting from the adsorption of multi-protonated organic acids onto carbon materials can be found in [7] and [8].

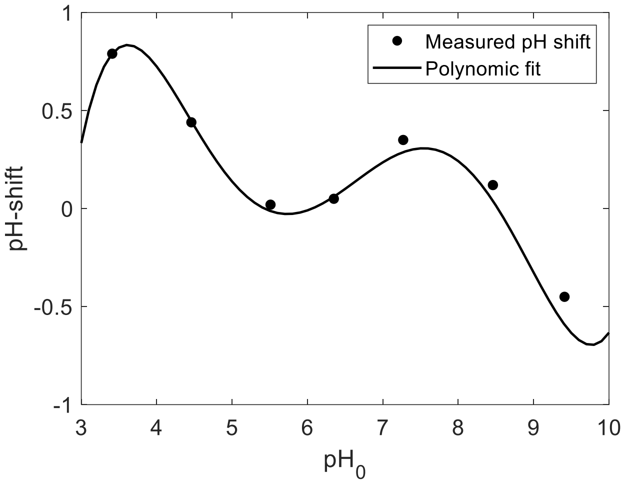

Figure S 6: As difference between the equilibrium pH and the initial pH value, the pH shift occurring during the potential free adsorption of maleic acid onto the carbon electrode Ecomite U is shown for initial pH values between 3.5 and 9.5 and a maleic acid concentration of 5 mM. The black line shows a polynomic fit (fifth degree polynomial) of the data points.

SI 1.5 Determination of the specific capacitance

The specific capacity of the carbon electrode was determined by cyclic voltammetry. The CV measurements were conducted in a three-electrode arrangement, including a piece of the carbon electrode as working electrode and a silver-silver chloride reference-electrode (Type RE-3VT from ALS, Tokyo, Japan). As counter electrode, a disc of a platinized, expanded titanium sheet with a diameter of electrode 18 mm, a mesh size of 1.5 mm, and a wire thickness of 1 mm was used. The resulting cyclic voltammogram of the second and third cycle is presented in Figure S 7.

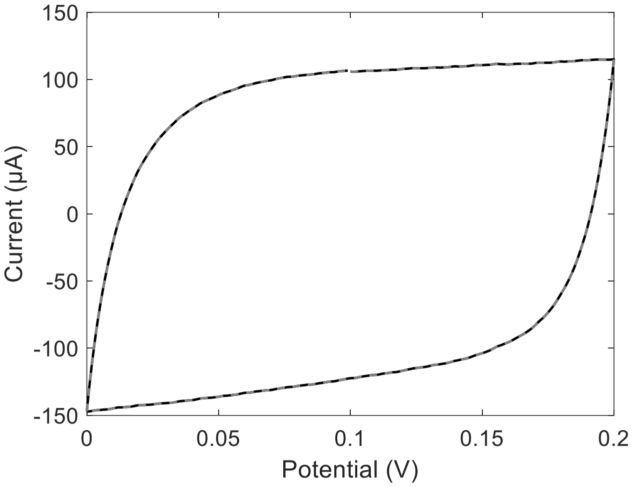

Figure S 7: Cyclic voltammogram for an Ecomite U electrode in 50 mmol/L NaCl solution and a scan rate of 0.1 mV/s. The second cycle of the experiment is marked by a black dashed line and the third cycle by a grey dashed line.

The high level of agreement between the two measurement cycles indicates a successful run with reliable results. To calculate the capacitance of the electrode, an equivalent circuit model consisting of a resistor with the resistance R_{setup}, and a capacitor with the capacity C_c, which were connected in series, was used. The parameters were determined by fitting the potential dependent current $I_c(U_{source})$ (equation (20)) to the experimentally determined currents.

$$I_c(U_{source}) = C_c \cdot v + (I_0 - C_c \cdot v) \cdot \exp\left(\frac{U_0 - U_{source}}{C_c \cdot v \cdot R_{setup}}\right) \tag{20}$$

With the scan rate v (0.1 mV/s), the applied voltage of the voltage source U_{source}, the initial voltage U_0 (0.1 V), which was determined by the open circuit potential, and the initial current I_0, which was calculated as quota of U_0 and R_{setup}. The fitting was conducted using the fittype function of Matlab.

By dividing the capacity by the electrode sample mass of 24.2 mg, a specific capacitance of 45 +/-0.16 F/g was determined with a coefficient of determination of 0.99.

SI 1.6 Adsorption capacities expressed in molar units

Figure S 8 shows the predicted electrosorption capacities for different maleic acid concentrations and different pH values. In contrast to Figure 3, the plots show the calculated molar amounts of adsorbed substance per gram of electrode mass. The lines were calculated with the herein presented model for an ideal constant voltage experiment with a voltage of 1.2 V.

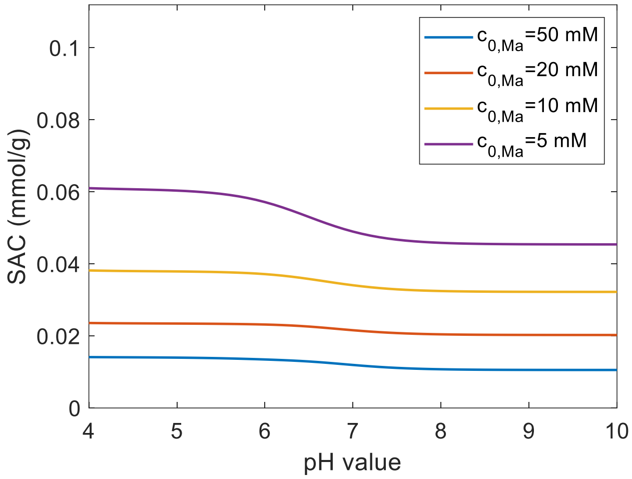

Figure S 8: The molar adsorption capacity predicted by the model, shows a slight decrease with rising pH values. This trend is shown for bulk concentrations of maleic acid of 5, 10, 20 and 50 mM, while an increasing bulk concentration leads to lower capacities.

A comparison to Figure 3 shows, that even though the charge efficiency is higher for increasing pH values, the amount of substance, that can be stored is slightly decreasing.

SI 1.7 Conductivity of maleic acid solutions

To evaluate the specific conductivities of the solutions, the equivalent conductivity listed in Table S 1 were used:

Table S 1: Equivalent conductivities of the ions present in the test solution [4]. **The conductivities were determined by titration of maleic acid with NaOH.*

Ion	$\Lambda\ \left(\frac{m^2}{\Omega \cdot mol}\right)$
Na^+	50.1
OH^-	198.6
H^+	349.8
$C_4O_4H_4$	0*
$C_4O_4H_3^-$	40*
$C_4O_4H_2^{2-}$	183*

For experimental validation of the accuracy of eq. (20) the conductivities of various maleic acid solutions were measured experimentally. For this the pH of maleic acid solutions with total concentrations of 5, 10, 20 and 50 mM was changed stepwise by titration (702 SM Titrino, Metrohm, Swiss) using a 2 M NaOH

(VWR Analytics, Germany). For each solution a volume of 1 L was provided and titrated with 0.05 mL/min (5 mM) or 0.2 mL/min (10, 20, 50 mM). For online monitoring a pH-Meter 765 Calimatic (Knick, Germany) with a SJ 114 (VWR, Germany) pH electrode and a conductivity meter (Type 703, Knick, Germany) with a CDC-314 (Radiometer Copenhagen, Denmark) conductivity measurement cell were used. Both probes were included in a loop in which the solution was pumped with 15 ml/min with a peristaltic pump (MasterFlex L/S, Cole-Parmer, USA). The resulting conductivity over pH is shown in comparison to the calculated conductivity in Figure S 9.

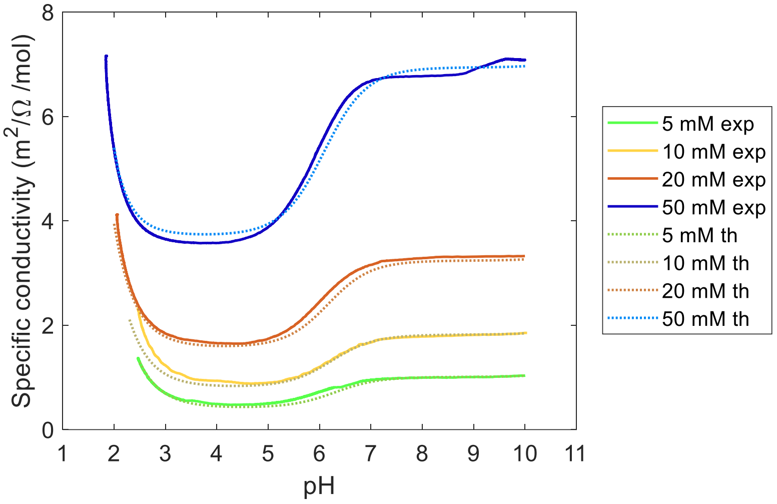

Figure S 9: Comparison between the measured conductivities (lines) of maleic acid for a pH range between pH 2 and 10 and the calculated conductivities (dotted lines) for concentrations of 5, 10, 20 and 50 mM. The shown titration curves resulted from a single experiment.

As shown in Figure S1, the deviation between the predicted and the measured conductivities is almost neglectable.

SI 1.8 Estimation of purification costs

In order to estimate the costs of a large-scale purification of maleic acid, experimentally determined quantities, such as SAC and charge efficiency, were transferred to a 10000 L fermentation process. For this purpose, it is assumed that a continuous fluid flow is pumped via a bypass loop from the fermenter through the CDI-unit, in order to keep the maleic acid concentration in the fermenter constant at 10 mmol/L. Thus, the amount of substance to be adsorbed from the solution equals the production rate of the fermentation process (1 g/L/h). The parameters used for the estimation are listed in Table S 2.

Table S 2: Parameters used to estimate the costs for isolation and purification of maleic acid by a CDI process. The parameters are grouped into the subsections: General parameters, parameters taken from the CDI experiments and parameters of the purification process.

Symbols	Description (unit)	Value
**General parameters**		
	Molecular weight (g/mol)	116
F	Faraday constant (C/mol)	96485
	Electricity price (€/kWh)	0.1
**CDI – Parameter (experiment)**		
ASAR	Average salt adsorption rate (mmol/g/min)	0.001
Λ	Charge efficiency	0.25
V_{Cell}	Voltage (V)	1.2
**Process Parameter (fermentation)**		
$Vol_{Fermentation}$	Fermenter volume (L)	10000
	Concentration (mmol/L)	10
	Production rate (g/L/h)	1
r	Production rate (mmol/L/h)	8.62

The necessary mass of the carbon electrodes per fermenter volume can be derived by dividing the production rate by ASAR and multiplying it with the fermenter volume, which results in a required mass of 1437 kg of carbon. To keep the conditions equal to the experiment, the fluid flow rate for fermentation can be estimated by dividing the electrode mass required in the industrial fermentation process by the mass of the carbon electrode of the lab scale experiment and multiplying it by the fluid flow rate of the experiment. This leads to a fluid flow rate for the fermentation process of 86.6 L/min. After defining the process parameters, the energy costs can be determined. The current to be applied results from equation (21), resulting from the charge of the amount of molecules adsorbed onto the carbon under consideration of the charge efficiency.

$$I = \frac{r \cdot F}{\Lambda} \cdot Vol_{Fermentation} = 9241.9\ A \tag{21}$$

With the current applied, the necessary power of 11090.2 W can be calculated as the product of the current and the voltage of 1.2 V. In combination with the electricity price, this leads to electricity costs of 1.11 € per hour or in relation to the mass of adsorbed maleic acid, 0.11 €/kg for the assumed production of 10 kg/h.

References

[1] Mortimer, C. E. and Müller, U., Eds., “19 Säure-Base-Gleichgewichte,” 13. Auflag., Georg Thieme Verlag, 2019.

[2] Davies, C. W., “397. The extent of dissociation of salts in water. Part VIII. An equation for the mean ionic activity coefficient of an electrolyte in water, and a revision of the dissociation constants of some sulphates,” *J. Chem. Soc.*, vol. 0, no. 0, p. 2093, Jan. 1938, doi: 10.1039/jr9380002093.

[3] Persat, A., Chambers, R. D., and Santiago, J. G., “Basic principles of electrolyte chemistry for microfluidic electrokinetics. Part I: Acid–base equilibria and pH buffers,” *Lab Chip*, vol. 9, no. 17, p. 2437, Sep. 2009, doi: 10.1039/b906465f.

[4] Teetz, T., *Experimentelle Elektrochemie*. De Gruyter, 2017.

[5] Hamann, C. H. and Vielstich, W., *Elektrochemie*, 4., vollst. Weinheim: Wiley-VCH, 2005.

[6] Hertwig, K., “Chemische Verfahrenstechnik : Berechnung, Auslegung und Betrieb chemischer Reaktoren.” De Gruyter, Berlin, 2018.

[7] Wagner, R., Bag, S., Trunzer, T., Fraga-García, P., *et al.*, “Adsorption of organic molecules on carbon surfaces: Experimental data and molecular dynamics simulation considering multiple protonation states,” *J. Colloid Interface Sci.*, 2021, doi: https://doi.org/10.1016/j.jcis.2020.12.107.

[8] Schewe, N., Wagner, R., Franzreb, M., and Thissen, P., “Role of the Hydration Shell in the pH-Dependent Adsorption of Maleic Acid,” *J. Phys. Chem. C*, vol. 125, no. 22, pp. 12305–12315, 2021, doi: 10.1021/acs.jpcc.1c01765.
